# Isorhamnetin Attenuates Isoproterenol-Induced Myocardial Injury by Reducing ENO1 (Alpha-Enolase) in Cardiomyocytes

**DOI:** 10.3390/antiox14050579

**Published:** 2025-05-11

**Authors:** Zhenli Guo, Shizhong Liu, Xianghong Hou, Xin Zhou, Yan Wang, Yi Rong, Xinzhi Li, Rui Yang, Ketao Ma

**Affiliations:** 1 The Key Laboratory of Xinjiang Endemic and Ethnic Diseases, Ministry of Education, Shihezi University Medical College, Shihezi 832002, China; zl_guo2025@163.com (Z.G.); 13345463523@163.com (S.L.); 15299949265@163.com (X.H.); zx19730804@163.com (X.Z.); wangyan_9011@shzu.edu.cn (Y.W.); rongyi0328@163.com (Y.R.); lixinzhi@shzu.edu.cn (X.L.); 2 Department of Physiology, Shihezi University Medical College, Shihezi 832002, China; 3 Department of Pathophysiology, Shihezi University Medical College, Shihezi 832002, China

**Keywords:** isorhamnetin, isoprenaline, myocardial injury, ENO1, glycolysis, oxidative stress

## Abstract

The protective effect of isorhamnetin on myocardial injury induced by isoproterenol (ISO) was investigated to identify the key targets and pathways involved, offering potential therapeutic insights for cardiovascular diseases. A myocardial injury model was established through intraperitoneal ISO injection, and the effects of isorhamnetin on apoptosis and oxidative stress in ISO-induced myocardial injury rats were assessed. Additionally, an ISO-induced H9c2 cell injury model was established to evaluate the impact of isorhamnetin on cellular damage. The transcriptomic sequencing of H9c2 cells was conducted to identify differentially expressed genes, followed by gene enrichment analysis. Intracellular glucose, lactate, and ATP levels were quantified, and the protein expression of key pathway targets ENO1, PPARα, and PGC-1α was analyzed via immunoblotting. Isorhamnetin improved cardiac function and morphological damage, reduced serum markers of cardiac injury, and exerted cardioprotective effects by regulating oxidative stress and inhibiting apoptosis. Compared to the ISO group, the glycolytic process—with ENO1 as a key target and the PPAR signaling pathway as the core regulator—was significantly suppressed in the isorhamnetin-pretreated group. Furthermore, isorhamnetin pretreatment reduced intracellular glucose and lactate levels while increasing ATP content in a concentration-dependent manner. These findings suggest that isorhamnetin protects the heart by inhibiting ENO1, activating the PPARα/PGC-1α signaling axis, reversing isoprenaline-induced metabolic shifts in H9c2 cells, suppressing glycolysis, and enhancing ATP release, thereby mitigating apoptosis and oxidative stress.

## 1. Introduction

For nearly three decades, cardiovascular diseases (CVDs) have been the leading cause of global mortality [1]. Recent reports have indicated that CVDs were responsible for approximately 20.5 million deaths in 2021, accounting for nearly one-third of all global fatalities [2]. Despite advancements in heart failure management, readmission and mortality rates remain alarmingly high. Isoprenaline (ISO)—a synthetic nonselective β-adrenergic agonist—exerts positive inotropic effects; yet, when administered in high doses, it can increase myocardial oxygen consumption and elevate myocardial injury markers, culminating in cardiac dysfunction [3]. CK-MB is one of the isoenzymes of creatine kinase (CK). When myocardial cells are damaged, CK-MB is released into the blood, such that an increase in its concentration is a sign of myocardial injury. cTnI is one of the subtypes of cardiac troponin (cTn), which exists only in cardiomyocytes and has high myocardial specificity. When myocardial cells are injured, cTnI is rapidly released into the blood and is the “gold standard” for diagnosing myocardial injury. Addressing ISO-induced cardiac dysfunction and exploring novel cardioprotective agents are of critical clinical significance.

Numerous studies have suggested that ISO-induced myocardial damage is linked to various pathophysiological mechanisms, including mitochondrial dysfunction [4], apoptosis [5], inflammation [6], oxidative stress [7], and disrupted energy homeostasis [8]. Bax, Bcl-2, and cleaved caspase-3 are recognized apoptotic markers. The upregulation of Bax and cleaved caspase-3 expression and the downregulation of Bcl-2 expression indicate an increase in apoptosis. ROS is a chemically active oxygen-containing molecule or free radical that can react with biomolecules, causing oxidative damage and serving as a biomarker of oxidative stress.

Enolase (ENO) is a type of highly conserved enzyme protein in prokaryotes and eukaryotes. It was first discovered to be involved in the glycolysis process, catalyzing the interconversion between 2-phosphoglyceric acid and phosphoenolpyruvic acid in glycolysis. Recent studies have shown that enolase—in addition to participating in the glycolytic process—has the characteristics of a “part-time protein”, with unique regional expression specificity and complex multifunctionality, and is involved in the pathophysiological processes of diseases such as tumors, infections, immunity, and metabolism [9,10]. Ji et al. demonstrated that the downregulation of ENO1 can suppress glycolysis, thereby reducing myocardial fibrosis after a heart attack [11]. However, the interplay between ENO1, apoptosis, and oxidative stress in cardiomyocytes during heart failure progression remains poorly understood.

As cardiovascular research advances, there has been a growing interest in the pharmacological and biological properties of compounds derived from traditional Chinese medicines [12]. Flavonoids have received considerable attention for their therapeutic potential [13,14]. Among these, isorhamnetin, also known as 3-methylquercetin and 3,5,7-trihydroxy-2-(4-hydroxy-3-methoxyphenyl)-4H-chromen-4-one, is a promising natural flavonoid compound extracted from ginkgo biloba and sea buckthorn [15]. Recent studies have emphasized its diverse biological activities, including cardiovascular protection [16,17], anti-apoptotic effects [18], and antioxidant properties [19]. However, the capacity of isorhamnetin to mitigate ISO-induced myocardial damage by restoring energy metabolism through glycolysis inhibition remains unexplored, and its underlying mechanisms are not fully understood.

The protective effects and molecular mechanisms of isorhamnetin against ISO-induced myocardial injury were investigated in this study. Both in vivo models of ISO-induced heart injury and in vitro models of ISO-induced H9c2 myocardial cell damage, coupled with transcriptomic analysis, were utilized in this research to provide a scientific foundation for the clinical application of therapeutic agents in the treatment of myocardial injury.

## 2. Materials and Methods

### 2.1. Materials

Isorhamnetin (Isor; B21554) was obtained from Shanghai Yuanye Biotechnology Co. (Shanghai, China). Isoproterenol hydrochloride (ISO; I5627) was purchased from Sigma-Aldrich (St. Louis, MO, USA). Primary antibodies for Bax (ab32503), Bcl-2 (ab182858), caspase-3 (ab184787), and ENO1 (ab227978) were sourced from Abcam (Cambridge, UK). Nrf2 (16396-1-AP), NQO1 (11451-1-AP), HO-1 (10701-1-AP), PPARα (66826-1-Ig), PGC-1α (66369-1-Ig), and β-actin (66009-1-Ig) antibodies were provided by Proteintech Biotechnology Co. (Wuhan, China). Secondary antibodies, goat anti-rabbit IgG (ZB-2306) and goat anti-mouse IgG (ZB-2305), were acquired from Zhong Shan-Golden Bridge Biological Technology Co. (Beijing, China). Kits for lactate dehydrogenase (LDH; A020-2-2), superoxide dismutase (SOD; A001-3-2), malondialdehyde (MDA; A003-1-2), glucose (GLU; F006-1-1), and lactic acid (LD; A019-2-1) were obtained from Nanjing Jiancheng Biotechnology Co., Ltd. (Nanjing, China). Creatine kinase isoenzyme (CK-MB; JL12296) and cardiac troponin I (cTnI; JL13014) kits were purchased from Shanghai Jianglai Industry Co., Ltd. (Shanghai, China). Cell viability was assessed using Cell Counting Kit 8 (CCK-8) detection kits from APExBIO Technology LLC (Shanghai, China). The Annexin V-FITC/PI Apoptosis Kit was procured from Multisciences Biotech Co. (Hangzhou, China). Dimethyl sulfoxide (DMSO), RIPA buffer, phenylmethylsulfonyl fluoride (PMSF), and an Oil Red O staining kit were purchased from Solarbio Science & Technology Co., Ltd. (Beijing, China).

### 2.2. Animals and Experimental Protocols

Male SD rats, weighing between 160 and 180 g, were obtained from Henan Skobes Biotechnology Co., Ltd. (Anyang, China). The rats were housed in the Animal Experimental Feeding Center at Shihezi University, where appropriate living conditions were provided. All animal experiments and procedures were approved by the Institutional Animal Care and Use Committee of Shihezi University, in compliance with the university’s ethical guidelines for animal experimentation. Myocardial injury was induced via the intraperitoneal injection of ISO (85 mg/kg) on two consecutive days, with a 24 h interval between doses. The rats were randomly assigned to five groups, each consisting of 8–10 animals, with the following treatment regimens:

Control group (Control): Normal saline was administered for nine consecutive days.

ISO group (ISO): Rats were given saline for the first seven days, followed by ISO (85 mg/kg) on days eight and nine via intraperitoneal injections.

Isorhamnetin low-dose group (ISO + Isor-L) and high-dose group (ISO + Isor-H): Isorhamnetin (5 mg/kg or 10 mg/kg) was administered intraperitoneally every 24 h from day 1 to day 7, followed by ISO (85 mg/kg) on days 8 and 9.

Propranolol group (ISO + Pro): Propranolol was administered intraperitoneally at a dose of 10 mg/kg daily during the first week, with ISO (85 mg/kg) given on days eight and nine.

The rats were anesthetized after a 24 h period following the final dose of ISO, and their thoracic cavities were rapidly exposed. Arterial blood was extracted from the abdominal aortas of rats. After standing for 30 min, it was centrifuged. The supernatant was aspirated to obtain serum, which was used for subsequent ELISA detection, and the heart was excised. The rat apical heart tissue was preserved in 4% paraformaldehyde and used to prepare paraffin sections, which were subsequently stained with HE, Masson, and TUNEL stains. The remaining portion was used to extract animal proteins for Western blotting.

### 2.3. Cell Culture and Treatments

H9c2 cells were obtained from the Cell Bank of the Chinese Academy of Sciences, Shanghai. These cells were cultured in DMEM supplemented with 10% fetal bovine serum and 1% penicillin–streptomycin. The cells were divided into several groups: a control group, an ISO group, an ISO + Isor (1.25 μM) group, an ISO + Isor (2.5 μM) group, and an ISO + Isor (5 μM) group. In the experimental setup, H9c2 cells were treated with isorhamnetin for 2 h, followed by exposure to ISO (140 μM) for 48 h.

### 2.4. Cardiac Injury-Associated Enzymes

To evaluate the cardiac function and myocardial injury of rats, we extracted the serum of rats from abdominal aortas and used ELISA kits to detect the activities of cTnI (cardiac troponin I) and CK-MB (Creatine kinase isoenzyme) in the serum. The samples were prepared according to the kit instructions and added to a 96-well plate. After the reaction was completed, the absorbance was measured with an enzyme-linked immunosorbent assay reader to evaluate the enzyme activities of cTnI and CK-MB.

### 2.5. Histopathological Study

The heart tissue was fixed in 4% paraformaldehyde and then embedded in paraffin and sectioned into 5 μm thick slices to evaluate the pathological changes and fibrosis degree of rat cardiac tissue. These sections underwent H&E and Masson staining. Stained slices were analyzed and photographed under a light microscope.

### 2.6. Terminal Deoxynucleotidyl Transferase dUTP Nick End Labeling (TUNEL)

The cardiac tissue segments were subjected to TUNEL staining to detect the apoptosis rate of cells, following the manufacturer’s instructions. The extent of myocardial cell death was quantified by calculating the ratio of TUNEL-positive nuclei to the total number of cardiac cells within the field of view.

### 2.7. Detection of Reactive Oxygen Species Using DHE Staining

H9c2 cells (2 × 10^5^) were seeded in 6-well plates with 2 mL of growth medium. Once the cells reached 60% confluence, they were treated with 140 μM ISO for 48 h. The culture medium was then replaced with a fresh medium containing 10% FBS. The cells were incubated with 25 μM DHE (Beyotime, Shanghai, China) for 25 min at 37 °C in the dark for reactive oxygen species (ROS) detection. Following incubation, the DHE-containing medium was discarded, and the wells were rinsed with 0.01 M PBS. Intracellular oxidative stress was assessed via fluorescence microscopy, and data were acquired using a FACS flow cytometer (BD Biosciences, Franklin Lakes, NJ, USA). The FlowJo V10 software was used to perform quantitative analysis of free radicals. Cell populations were gated based on size and granularity (SSC-A vs. FCS-A), and the fluorescence intensity of the gated cells was analyzed to evaluate the antioxidant capacity of the enzymes.

### 2.8. Lactate Dehydrogenase Assay

Supernatants from the cell culture were collected, and lactate dehydrogenase (LDH) release was assessed to determine cellular damage, following the kit’s protocol. Absorbance was measured at 450 nm.

### 2.9. Cell Viability Assay

Cells were exposed to varying concentrations (0, 40, 80, 100, 120, 140, 160, or 180 μM) of ISO in the culture medium for 24, 48, or 72 h. After incubation, the medium was replaced with a fresh medium containing CCK-8 reagent (APExBio, Houston, TX, USA). Following an additional 2–3 h incubation, absorbance was measured at 450 nm using a Thermo Fisher microplate reader (Thermo Fisher, Shanghai, China). Cell viability was determined by comparing the absorbance values to those of untreated control cells.

### 2.10. Flow Cytometry Assay: Annexin-V/PI Assay for Apoptosis

Apoptosis was assessed using the Annexin V-FITC/PI Apoptosis Detection Kit (Multi Sciences, Hangzhou, China). Cells were incubated with 5 μL of Annexin V-FITC and 10 μL of PI solution and were then analyzed with flow cytometry. The percentage of apoptotic cells was calculated using FlowJo V10 software.

### 2.11. Western Blot Analysis

RIPA and protease inhibitors were mixed in a ratio of 100:1 to prepare the lysis buffer. Cardiac tissue or cells were lysed using lysis buffer, and the protein concentration was quantified via the BCA assay kit. Proteins were separated with 10% SDS-PAGE and transferred onto PVDF membranes. Membranes were blocked with 5% non-fat milk for 2 h at room temperature and were then incubated with primary antibodies overnight at 4 °C. Afterward, membranes were incubated with horseradish peroxidase-conjugated secondary antibodies (diluted 1:20,000) for 2 h. Chemiluminescent signals were detected using an ECL kit (Biosharp, Hefei, China) and captured via a chemiluminescence imaging system. Images were saved for further quantitative analysis, using β-actin as the internal control.

### 2.12. Gene Set Enrichment Analysis (GSEA)

H9c2 cells were grouped into control, ISO, and ISO + Isor treatment conditions. RNA was extracted from H9c2 cells using TRIzol reagent (Thermo Fisher, Shanghai, China), and the samples were sent to NovelBio (Shanghai, China) for further analysis.

### 2.13. RNA Extraction and RNA Sequencing Analysis

Transcriptomic data were processed using the OmicShare platform “https://www.omicshare.com/” (accessed on 14 October 2023), utilizing the dynamic GSEA enrichment tool and selecting “Homo sapiens (GRCh38.p13)” for species. The Molecular Signature Database (MSigDB) was employed to identify hallmark gene sets, facilitating the identification of key targets and mechanisms through which isorhamnetin modulates ISO-induced myocardial injury, based on the expression profiles of differentially expressed genes (DEGs).

### 2.14. Docking Analysis

The molecular docking study was conducted using AutoDockTools 1.5.7 to investigate the binding interactions between isorhamnetin and its primary target. The 3D structure of isorhamnetin was retrieved from the PubChem database https://pubchem.ncbi.nlm.nih.gov/ (accessed on 2 November 2023), and the structure of the primary target protein was obtained from the PDB database https://www.rcsb.org/ (accessed on 2 November 2023). The target protein’s structure was refined using PyMol 2.6.0. Isorhamnetin was set as a ligand for semiflexible docking with the target using AutoDockTools. Binding interactions and modes were analyzed, and the configurations with the lowest binding energies were visualized.

### 2.15. RNA Knockdown

The ENO1-targeted small interfering RNA (siRNA) was purchased from JTS Scientific, Wuhan, China. A non-specific siRNA was used as the negative control. Cells were transfected with the siRNA using a reagent provided by Gene Pharma (JTS Scientific, Wuhan, China) and cultured for 48 h according to the manufacturer’s protocol. Transfection efficacy was assessed using Western blot analysis. The specific sequence for ENO1 was 5′-GAGCAGAGGUUUACCACAATT-3′, and the complementary sequence was 3′-UUGUGGUAAACCUCUGCUCTT-5′.

### 2.16. Statistical Analysis

Statistical analysis was performed using the GraphPad Prism 8.0 software. When quantitative data follow a normal distribution, it is described by mean ± standard deviation. The one-way analysis of variance (ANOVA) method is used for comparison among multiple groups. If the ANOVA results show significant differences and satisfy homogeneity of variance, the least significant difference (LSD) method is used for multiple comparisons between groups. If the results of the analysis of variance are significantly different and do not satisfy homogeneity of variance, Dunn’s multiple comparisons should be used. When the quantitative data do not follow a normal distribution, the median and interquartile range is used for description, and non-parametric tests are used for comparison among multiple groups. Statistical significance was defined as *p* < 0.05.

## 3. Results

### 3.1. Isorhamnetin Ameliorates ISO-Induced Cardiac Dysfunction in a Rat Model

#### 3.1.1. Evaluating Cardiac Function via Echocardiography

**Figure 1:**
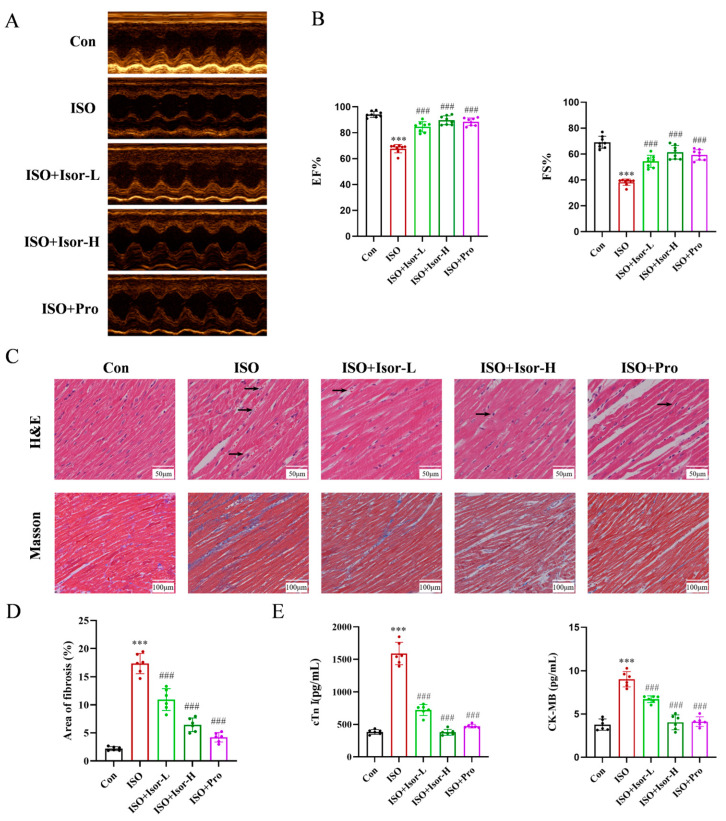
Isorhamnetin ameliorates ISO-induced cardiac dysfunction in rat heart tissue. Effects of isorhamnetin on cardiac function indices. ISO: isoproterenol (85 mg/kg); ISO + Isor-L: isorhamnetin low-dose group (5 mg/kg); ISO + Isor-H: isoproterenol high-dose group (10 mg/kg); ISO + Pro: propranolol group (10 mg/kg). (**A**) M-mode echocardiograms of different groups. (**B**) Echocardiographic assessment of ejection fraction (EF%) and fractional shortening (FS%) for quantification of impaired cardiac function, *n* = 8. (**C**) Representative H&E and Masson’s trichrome-stained images of rat myocardial tissue from each group (scale bar = 50 or 100 μm). The black arrows refer to the lesions of heart failure, including wavy myocardium, cell edema, mild vacuolar degeneration, sarcoplasmic condensation of some cardiomyocytes, nuclear pyknosis, inflammatory cell infiltration, interstitial edema in varying degrees, and even bleeding. (**D**) Statistical analysis of the fibrosis area in myocardial tissue across groups, *n* = 6. (**E**) Effect of isorhamnetin on the enzyme activities of cTnI and CK-MB in ISO-treated rats, *n* = 6. Data are expressed as means ± SDs. *** *p* < 0.001 vs. the control group; ### *p* < 0.001 vs. the ISO group.

Echocardiography was employed to evaluate cardiac function in rats. As shown in Figure 1A, rats administered isoprenaline via intraperitoneal injection exhibited significant cardiac dysfunction compared to the control group. However, treatment with isorhamnetin and the positive control, propranolol, notably improved cardiac performance. Left ventricular ejection fraction (EF%) and fractional shortening (FS%) values, measured via echocardiography, are presented in Figure 1B. EF% and FS% in the ISO model group were significantly lower than in the control group, whereas those in the isorhamnetin and propranolol pretreated groups were considerably elevated. These results indicate that isorhamnetin alleviates ISO-induced cardiac damage. Furthermore, the enhancement of EF% and FS% by isorhamnetin was dose-dependent, with its protective effect paralleling that of the positive control drug, propranolol. Clinically, propranolol has a protective effect on the heart. In this experiment, propranolol was used as a protective positive control drug to compare the cardioprotective effect of isorhamnetin.

#### 3.1.2. Isorhamnetin Alleviates ISO-Induced Myocardial Histopathological Damage and Myocardial Fibrosis in Rats

The pathological histological changes in cardiac tissue across groups were assessed using HE staining. As illustrated in Figure 1C, both isorhamnetin and the positive control, propranolol, mitigated ISO-induced pathological alterations in the heart. In normal cardiac tissue, myocardial cells displayed a uniform shape with distinct longitudinal and transverse striations, and myofibrils were neatly organized. In contrast, the ISO model group exhibited varying degrees of cell swelling, vacuolar degeneration, cytoplasmic coagulation, dense nuclei, disordered myocardial fibers, inflammatory cell infiltration, and occasional hemorrhage. Treatment with isorhamnetin and propranolol led to substantial improvements in myofiber alignment and reduced interstitial edema, with the high-dose isorhamnetin group showing a cellular structure closely resembling that of the normal group. To assess fibrous tissue formation, Masson staining was performed. The ISO group displayed marked fibrosis compared to the control, whereas myocardial fibrosis was progressively reduced in the isorhamnetin and propranolol treatment groups. The fibrosis area of the Masson staining results was calculated and statistically analyzed, as shown in Figure 1D. These results suggest that isorhamnetin effectively prevents ISO-induced myocardial fibrosis. As shown in Figure 1E, serum levels of CK-MB and cTnI were significantly elevated in the ISO model group, indicating myocardial damage. However, these markers were markedly reduced in the ISO + Isor-5 mg/kg, ISO + Isor-10 mg/kg, and ISO + Pro-10 mg/kg pre-treatment groups, demonstrating a dose-dependent attenuation of myocardial injury. These results indicate that isorhamnetin significantly alleviates ISO-induced myocardial injury in rats.

### 3.2. Isorhamnetin Reduces ISO-Induced Apoptosis and Oxidative Stress in Rats

#### 3.2.1. Isorhamnetin Alleviates ISO-Induced Myocardial Apoptosis in Rats

**Figure 2:**
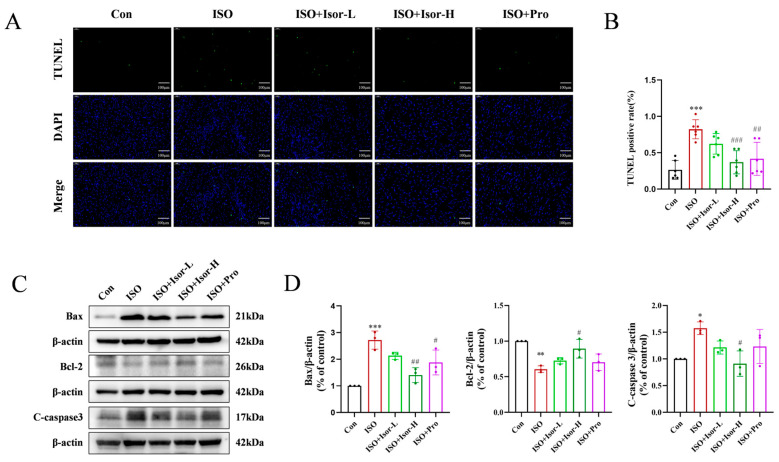
Isorhamnetin ameliorates ISO-induced apoptosis in rat heart tissue. (**A**) Effect of isorhamnetin on ISO-induced myocardial apoptosis, as shown by TUNEL staining. (**B**) Statistical analysis of TUNEL assay results for each group, *n* = 6. (**C**,**D**) Effects of isorhamnetin on ISO-induced expression of Bcl-2, Bax, and cleaved caspase-3 proteins in the rat myocardium, *n* = 3. Data are expressed as means ± SDs. * *p* < 0.05, ** *p* < 0.01, *** *p* < 0.001 vs. the control group; # *p* < 0.05, ## *p* < 0.01, ### *p* < 0.001 vs. the ISO group.

TUNEL fluorescence staining was employed to assess myocardial cell apoptosis. The ISO model group exhibited a significantly higher number of apoptotic cardiomyocytes compared to the control group (Figure 2A,B). In contrast, both the high-dose isorhamnetin and propranolol pretreatment groups demonstrated a marked reduction in apoptotic cardiomyocyte count relative to the model group. Immunoblotting data revealed that the ISO model group showed a significant decrease in the anti-apoptotic protein Bcl-2 compared to the control group, accompanied by an increase in the pro-apoptotic proteins Bax and cleaved caspase-3. Treatment with isorhamnetin and propranolol led to a substantial reduction in Bax and cleaved caspase-3 levels, along with an elevation in Bcl-2 expression (Figure 2C,D). Notably, higher concentrations of isorhamnetin exerted a more pronounced effect on apoptosis-related proteins than the positive control, propranolol.

#### 3.2.2. Isorhamnetin Alleviates ISO-Induced Myocardial Oxidative Stress in Rats

**Figure 3:**
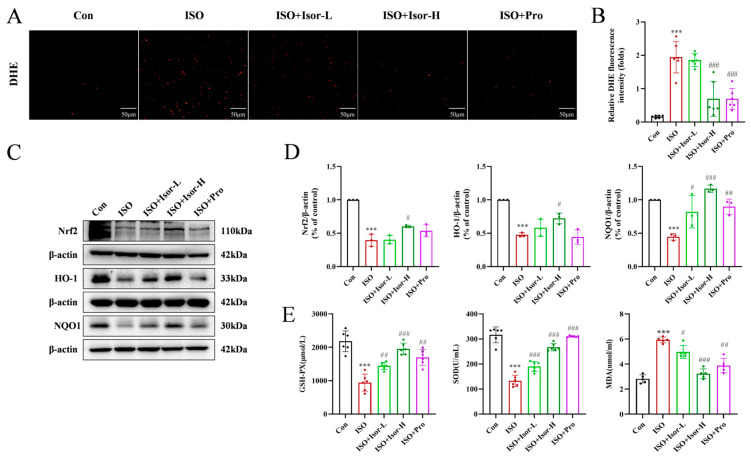
Isorhamnetin ameliorates ISO-induced oxidative stress in the rat heart tissue. (**A**,**B**) Effects of isorhamnetin on ISO-induced myocardial oxidative stress in rats, as shown by DHE staining, *n* = 6. (**C**,**D**) Effects of isorhamnetin on ISO-induced Nrf2, HO-1, and NQO1 protein expression in the rat myocardium, *n* = 3. (**E**) Effects of isorhamnetin on the activities of GSH-PX, SOD, and MDA enzymes in ISO-treated rats, *n* = 6. Data are expressed as means ± SDs. *** *p* < 0.001 vs. the control group; # *p* < 0.05, ## *p* < 0.01, ### *p* < 0.001 vs. the ISO group.

ROS levels in the cardiac tissue of rats were assessed using DHE staining. As shown in Figure 3A,B, the myocardium of the ISO model group exhibited more intense and widespread red fluorescence compared to the control group, indicating elevated ROS levels and substantial oxidative stress. In contrast, ROS levels were significantly reduced in the hearts of the ISO + Isor-5 mg/kg, ISO + Isor-10 mg/kg, and ISO + Pro-10 mg/kg groups, suggesting that isorhamnetin effectively mitigates oxidative stress in rat hearts.

The expression of oxidative stress-related proteins Nrf2, HO-1, and NQO1 was evaluated in rat heart tissues through immunoblotting. As shown in Figure 3C,D, these proteins were detectable in the cardiac tissues. Compared to the control group, the ISO model group exhibited a significant reduction in the expression of Nrf2, HO-1, and NQO1. In contrast, pretreatment with high doses of isorhamnetin and propranolol resulted in a marked increase in HO-1 and NQO1 expression in the heart tissues.

MDA is the highly oxidative product of oxidative stress. GSH and SOD are the antioxidant markers. The serum levels of GSH-PX, SOD, and MDA were measured to assess systemic oxidative stress. As shown in Figure 3E, the ISO group exhibited significantly reduced SOD and GSH-PX activities and elevated MDA levels compared to the control group, indicating increased oxidative stress following ISO administration. In contrast, the ISO + Isor-5 mg/kg, ISO + Isor-10 mg/kg, and ISO + Pro-10 mg/kg groups displayed significantly lower MDA levels and notably higher SOD and GSH-PX activities compared to the ISO group. These results demonstrate that isorhamnetin effectively alleviates ISO-induced myocardial oxidative stress in rats.

### 3.3. Isorhamnetin Increases Cell Viability and Alleviates Myocardial Injury in ISO-Injured H9c2 Cells

**Figure 4:**
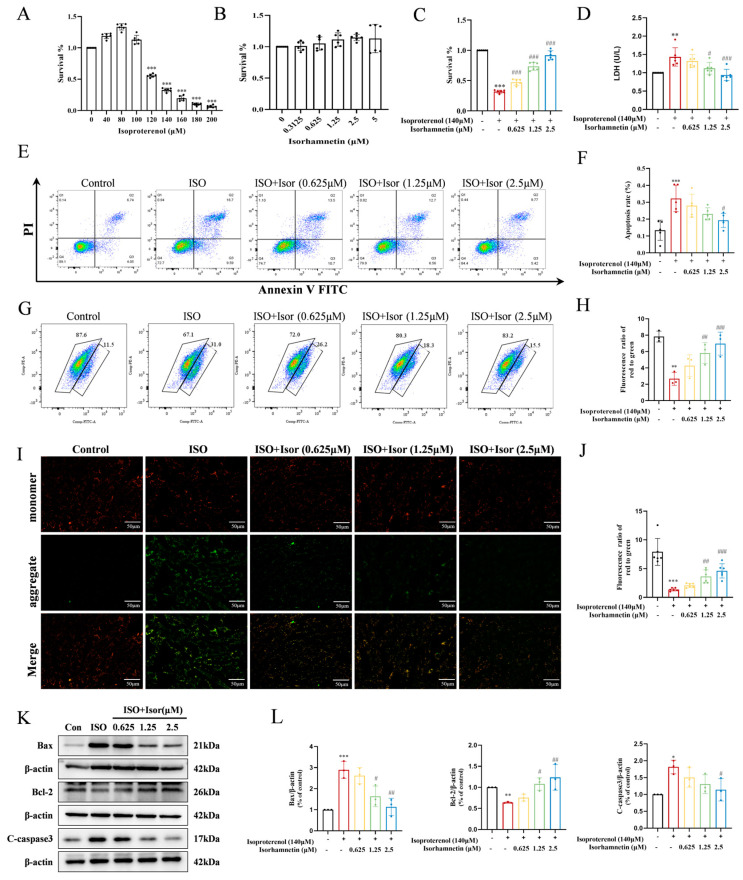
Isorhamnetin ameliorates ISO-induced H9c2 cardiomyocyte injury. (**A**) Effect of ISO on the survival rate of H9c2 cardiomyocytes, *n* = 6. (**B**) Effects of different concentrations of isorhamnetin on the survival rate of H9c2 cells, *n* = 6. (**C**) Effect of isorhamnetin on the survival rate of ISO-treated H9c2 cells, *n* = 6. (**D**) Effect of isorhamnetin on LDH activity in ISO-treated H9c2 cells, *n* = 6. (**E**,**F**) Effects of isorhamnetin on ISO-induced apoptosis in H9c2 cells, assessed by flow cytometry, *n* = 5. (**G**,**H**) JC-1 staining combined with flow cytometry analysis, *n* = 3. (**I**,**J**) JC-1 staining observed under an inverted microscope, *n* = 6. (**K**,**L**) Effects of isorhamnetin on ISO-induced protein expression of Bax, Bcl-2, and cleaved caspase-3 in H9c2 cells, as detected via Western blotting, *n* = 3. Data are expressed as means ± SDs. * *p* < 0.05, ** *p* < 0.01, *** *p* < 0.001 vs. the control group; # *p* < 0.05, ## *p* < 0.01, ### *p* < 0.001 vs. the ISO group.

The CCK-8 assay was employed to assess the viability of H9c2 cells, enabling the determination of optimal concentrations for ISO and isorhamnetin and the exposure duration. As shown in Figure 4A, after 48 h of exposure to ISO at concentrations ranging from 0 to 200 μM, cell viability increased with an ISO concentration of up to 80 μM. However, higher ISO concentrations resulted in reduced cell viability, with a significant decrease observed at 140 μM, establishing this concentration as the threshold for inducing cellular damage in subsequent experiments.

To evaluate the potential toxicity of isorhamnetin in H9c2 cells, various concentrations (0, 0.3125, 0.625, 1.25, 2.5, and 5 μM) were tested using the CCK-8 assay. Figure 4B demonstrates that isorhamnetin concentrations up to 5 μM did not significantly affect cell survival after 48 h, suggesting its non-toxic nature at these concentrations. Based on these results, concentrations of 0.625, 1.25, and 2.5 μM were selected for pre-treatment. Cells were pretreated with these concentrations of isorhamnetin, followed by 140 μM ISO treatment for 48 h, as shown in Figure 4C. The results indicate that isorhamnetin effectively mitigates the reduction in cell viability caused by ISO. Additionally, the LDH assay results (Figure 4D) reveal a significant increase in intracellular LDH levels after ISO treatment, reflecting myocardial cell damage. In contrast, the LDH levels in the isorhamnetin-treated groups, at low, medium, and high concentrations, were progressively lower than those in the ISO model group, suggesting that isorhamnetin provides protective effects against ISO-induced cardiomyocyte injury.

### 3.4. Isorhamnetin Reduces ISO-Induced Apoptosis and Oxidative Stress In Vitro

#### 3.4.1. Isorhamnetin Reduces ISO-Induced Apoptosis in H9c2 Cells

Apoptosis in H9c2 cardiomyocytes was evaluated via Annexin V-FITC/PI dual staining and flow cytometry. The model group exhibited a markedly higher apoptotic cell count in the Q2 + Q3 region compared to the control group (Figure 4E,F). In contrast, the high-dose isorhamnetin-pretreated group showed a significantly lower percentage of apoptotic cells, suggesting that isorhamnetin mitigates ISO-induced cardiomyocyte apoptosis. The underlying mechanism of ISO-induced apoptosis was further investigated using JC-1 staining and flow cytometry, with results shown in Figure 4G–J. ISO treatment notably reduced mitochondrial membrane potential in H9c2 cells relative to controls, whereas pretreatment with 0.625, 1.25, and 2.5 μM isorhamnetin restored mitochondrial membrane potential in a concentration-dependent manner. The expression of key apoptosis-related proteins—Bax, cleaved caspase-3, and Bcl-2—was examined through immunoblotting to assess the impact of isorhamnetin on apoptosis. In comparison to the controls, the ISO group displayed significantly elevated levels of the pro-apoptotic proteins Bax and cleaved caspase-3, alongside a decrease in the anti-apoptotic protein Bcl-2. Pretreatment with isorhamnetin, particularly at 2.5 μM, led to a notable reduction in Bax and cleaved caspase-3 levels while enhancing Bcl-2 expression (Figure 4K,L). These results confirm that isorhamnetin effectively attenuates ISO-induced cardiomyocyte apoptosis.

#### 3.4.2. Isorhamnetin Reduces ISO-Induced Oxidative Stress in H9c2 Cells

**Figure 5:**
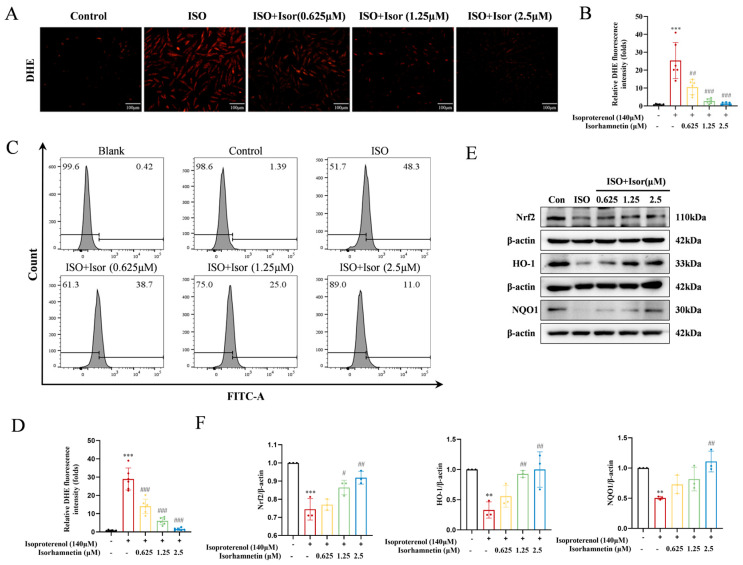
Isorhamnetin attenuates ISO-induced oxidative stress in H9c2 cells. (**A**,**B**) DHE fluorescence in each group, *n* = 6. (**C**,**D**) DHE combined with flow cytometry data, *n* = 6. (**E**,**F**) Effects of isorhamnetin on the ISO-induced protein expression of Nrf2, HO-1, and NQO1 in H9c2 cells, as detected via Western blotting, *n* = 3. Data are expressed as means ± SDs. ** *p* < 0.01, *** *p* < 0.001 vs. the control group; # *p* < 0.05, ## *p* < 0.01, ### *p* < 0.001 vs. the ISO group.

As illustrated in Figure 5A,B, DHE fluorescence was utilized to assess the impact of isorhamnetin on ROS levels in ISO-treated H9c2 cardiomyocytes. The model group exhibited a significantly higher number of cells with red fluorescence compared to the control group. However, pretreatment with isorhamnetin at concentrations of 0.625, 1.25, and 2.5 μM led to a dose-dependent reduction in the number of cells showing red fluorescence relative to the ISO group. The fluorescence intensity in the ISO group was markedly elevated compared to the control group, indicating a significant increase in ROS levels in H9c2 cardiomyocytes. In contrast, the fluorescence intensity in cells pretreated with various concentrations of isorhamnetin progressively decreased, reflecting a reduction in ROS content, with the effect becoming more pronounced at higher concentrations of isorhamnetin (Figure 5C,D). As shown in Figure 5E, the expression of Nrf2, HO-1, and NQO1 was lower in the ISO group compared to controls. In contrast, isorhamnetin pretreatment induced a concentration-dependent increase in the expression of these antioxidant markers relative to the model group (Figure 5E,F). These results suggest that isorhamnetin effectively mitigates ISO-induced oxidative stress in H9c2 cells.

### 3.5. Transcriptome Sequencing Enrichment Reveals the Inhibition of the Glycolytic Pathway After Isorhamnetin Treatment, with ENO1 as a Key Target, in H9c2 Cells

**Figure 6:**
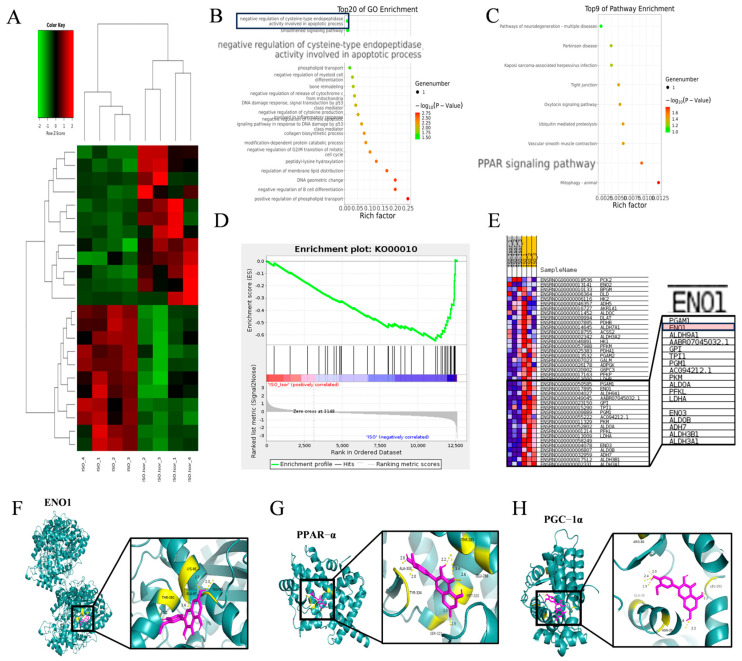
Transcriptome sequencing revealed the inhibition of the glycolytic pathway after isorhamnetin treatment, with ENO1 as a key target, in H9c2 cells. (**A**) RNA sequencing results showing differential gene expression in ISO-induced H9c2 cardiomyocytes treated with isorhamnetin. (**B**,**C**) Z-score bubble chart of KEGG and GO enrichment analyses of the DEGs identified via transcriptomics (upward normalization). (**D**) GSEA enrichment plot for the glycolytic metabolism gene set. (**E**) Gene expression clustering plot for the glycolytic metabolism gene set. (**F**–**H**) Molecular docking patterns of isorhamnetin with ENO1, PPARα, and PGC-1α molecules.

To investigate the regulatory effects of isorhamnetin on ISO-induced injury in H9c2 cardiomyocytes, high-throughput transcriptomic sequencing was performed to analyze gene expression profiles in control, ISO, and ISO + Isor (1.25 μM) groups. The analysis identified 7560 DEGs compared to the control group. Following isorhamnetin pretreatment, 11 genes were downregulated and 12 were upregulated relative to the ISO group. Figure 6A displays a heatmap of DEGs post-isorhamnetin treatment, with green indicating downregulation and red representing upregulation. GO enrichment analysis emphasized the role of the negative regulation of cysteine-type endopeptidases in apoptosis (Figure 6B), while KEGG pathway analysis highlighted the significance of the PPAR pathway (Figure 6C). GSEA of the DEGs revealed significant suppression of the glycolytic gene set, with an enrichment score (ES) of −0.6463721 and a false discovery rate (FDR) of 0.001 (Figure 6D). The gene expression clustering plot for glycolytic metabolism showed that *ENO1* was significantly downregulated, with a Log2FC of −0.676 and an FDR of 0 (corrected *p* < 0.001), suggesting *ENO1*′s pivotal role in glycolytic metabolism after isorhamnetin treatment (Figure 6E). Molecular docking simulations were carried out to assess the interaction between isorhamnetin and ENO1, PPARα, and PGC-1α, with the binding stability determined using docking scores. Figure 6F–H illustrate the binding patterns of isorhamnetin with ENO1, PPARα, and PGC-1α, showing binding energies below −1.2 kcal/mol, indicating robust binding. The lowest binding energies for isorhamnetin with ENO1, PPARα, and PGC-1α were −4.54, −7.16, and −8.05 kcal/mol, respectively, confirming isorhamnetin’s strong binding affinity for these molecules.

### 3.6. Isorhamnetin Downregulates ENO1 Expression, Reverses the ISO-Induced Increase in Intracellular Glycolysis, and Increases ATP Content in H9c2 Cells

**Figure 7:**
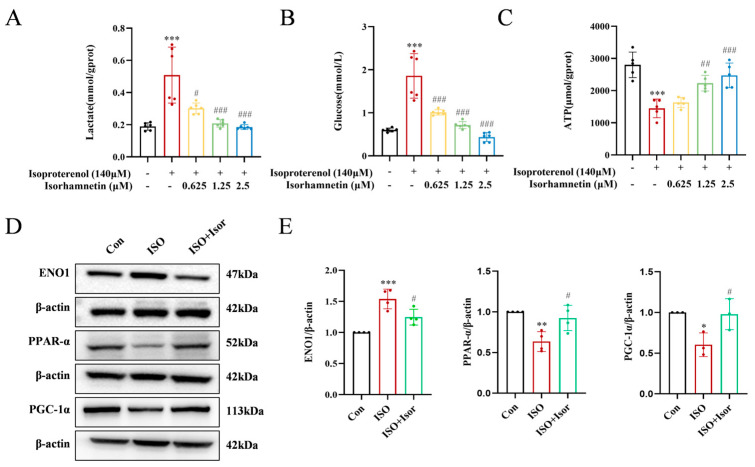
Isorhamnetin reversed the ISO-induced increase in intracellular glycolytic processes and increased ATP content in H9c2 cells. (**A**–**C**) Statistical graphs showing lactate, glucose, and ATP contents, *n* = 5, 6. (**D**,**E**) Immunoblotting bands displaying the protein expression of ENO1, PPAR-α, and PGC-1α in cells, *n* = 3, 4. Data are expressed as means ± SDs. * *p* < 0.05, ** *p* < 0.01, *** *p* < 0.001 vs. the control group; # *p* < 0.05, ## *p* < 0.01, ### *p* < 0.001 vs. the ISO group.

The transcriptomic analysis highlighted the pivotal role of the glycolytic pathway in ISO-induced cardiac injury. Significant alterations in glucose, the primary substrate for glycolysis, and lactate, its end product, were observed (Figure 7A,B). The ISO model group exhibited markedly higher levels of intracellular glucose and lactate compared to the control group. These levels were progressively reduced with the increase in isorhamnetin concentrations, suggesting that ISO treatment enhances glycolysis, while isorhamnetin inhibits this process. Regarding energy metabolism, glycolytically derived ATP levels were notably lower than those generated via fatty acid metabolism. As shown in Figure 7C, ATP levels in the ISO-treated group were significantly reduced compared to controls, and isorhamnetin mitigated the ISO-induced decrease in the cytoplasmic ATP content. To investigate further, immunoblotting was performed to assess the expression of ENO1, a key glycolytic enzyme, as well as PPARα and PGC-1α, key regulators in the PPAR signaling pathway, in H9c2 cells across control, ISO, and ISO + Isor (1.25 μM) groups. Figure 7D,E illustrate that ISO treatment led to a significant upregulation of ENO1 and downregulation of PPARα and PGC-1α expression. In contrast, isorhamnetin pretreatment reversed these changes, reducing ENO1 expression while enhancing PPARα and PGC-1α levels. These results indicate that isorhamnetin effectively counteracts ISO-induced glycolysis and exerts a protective effect on cardiac function.

### 3.7. Isorhamnetin Exerts Cardioprotective Effects by Inhibiting ENO1, Activating the PPARα/PGC-1α Signaling Axis, Reversing Isoprenaline-Induced Conversion of H9c2 Energy Metabolism Substrate Levels, Inhibiting Glycolysis, and Increasing ATP Release, Thereby Attenuating Apoptosis and Oxidative Stress

**Figure 8:**
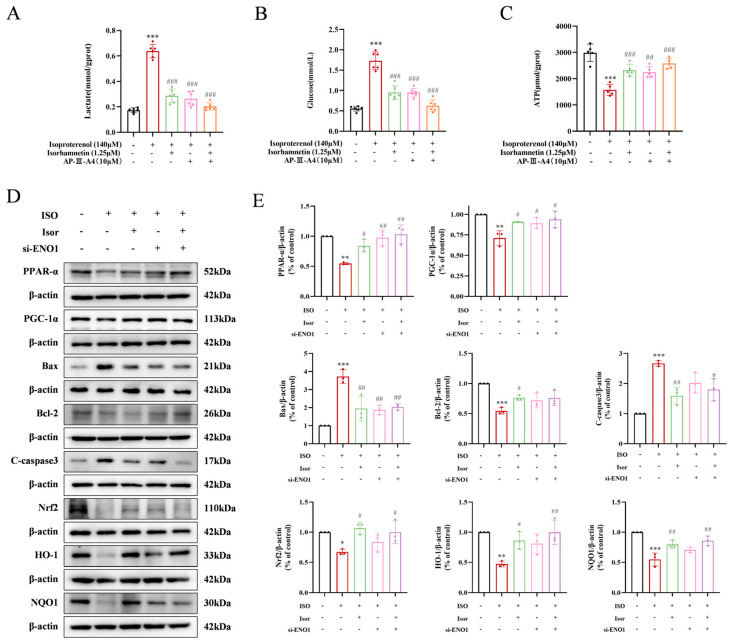
Isorhamnetin inhibits ENO1, activates the PPARα/PGC-1α signaling axis, reverses the transformation of H9c2 energy metabolism substrates induced by isoproterenol, inhibits glycolysis, increases ATP release, alleviates apoptosis and oxidative stress, and protects against cardiac injury. (**A**–**C**) Statistical graphs showing lactate, glucose, and ATP contents, *n* = 5, 6. (**D**,**E**) Western blot analysis of apoptosis, oxidative stress, and PPAR-signaling-pathway-related protein expression. β-actin was used as a control, *n* = 3. Data are expressed as means ± SDs. * *p* < 0.05, ** *p* < 0.01, *** *p* < 0.001 vs. the control group; # *p* < 0.05, ## *p* < 0.01, ### *p* < 0.001 vs. the ISO group.

To investigate the cardioprotective potential of isorhamnetin, ENO1 inhibition was employed using AP-III-a4, a non-substrate analog that binds to enolase and inhibits its activity in H9c2 cells. Glucose, the primary glycolytic substrate, and lactate, a glycolytic byproduct, were measured as shown in Figure 8A,B. The ISO model group exhibited significantly elevated intracellular glucose and lactate levels compared to the control group. In contrast, the AP-III-a4 inhibitor group displayed markedly reduced glucose and lactate levels, mirroring the protective effects of isorhamnetin. The combined treatment of AP-III-a4 and isorhamnetin further decreased these levels. As illustrated in Figure 8C, intracellular ATP content was significantly reduced in the ISO-treated group compared to controls. AP-III-a4 treatment mitigated the ISO-induced decline in ATP, and the ATP level was notably increased when both AP-III-a4 and isorhamnetin were used in combination.

To further explore the role of ENO1 in myocardial damage, siRNA targeting ENO1 was introduced into H9c2 cells, and knockdown efficiency was confirmed with Western blot analysis. The expression of apoptosis and oxidative stress markers was also assessed. The results showed that silencing ENO1 in H9c2 cells produced effects similar to isorhamnetin treatment. Specifically, ENO1 suppression reduced the expression of pro-apoptotic markers Bax and cleaved caspase-3 while enhancing the anti-apoptotic protein Bcl-2. Additionally, the increased expression of oxidative-stress-related markers, including Nrf2, HO-1, and NQO1, was observed (Figure 8D,E). These results collectively highlight the critical role of ENO1 in mediating cardiomyocyte injury.

## 4. Discussion

ISO, a β-adrenergic receptor agonist, plays a pivotal role in clinical conditions such as myocardial infarction, arrhythmia, heart failure, and stress-related heart diseases [8,20]. Propranolol is a non-selective beta-1 and beta-2 adrenergic receptor blocker, which can affect the function of adrenergic neurons and the sensitivity of blood pressure-regulating receptors in the central nervous system. It can competitively counteract the effect of isoproterenol [21,22]. In this study, propranolol was selected as the positive control. Thus, identifying more effective therapeutic strategies and novel targets for intervention is essential. Isorhamnetin, a natural flavonoid [23], has garnered attention due to its diverse biological activities. Recent studies highlight its potential as an anti-apoptotic, antioxidant, and anti-inflammatory agent, positioning it as a promising candidate for cardiovascular disease therapy [24]. For example, Xu et al. demonstrated that isorhamnetin could ameliorate the myocardial apoptosis and oxidative stress induced by ischemia–reperfusion in SD rats [25]. These findings suggest that isorhamnetin may offer protective benefits against ISO-induced myocardial injury, prompting further experimental investigation into this hypothesis.

Apoptosis is a regulated form of cell death that facilitates the efficient removal of damaged cells, such as those affected by DNA damage or developmental processes [26]. Endogenous apoptosis is primarily governed by mitochondrial function [27,28]. Previous studies have indicated that the inhibition of Bax activity and promotion of Bcl-2 expression could confer a direct cardioprotective effect in acute myocardial infarction [18,29,30]. In the present study, isorhamnetin inhibited Bax expression, promoted Bcl-2 expression, and reduced Caspase-3 activation, thereby exerting an anti-apoptotic effect.

Increased oxidative stress is recognized as a common contributor to cardiovascular diseases [31]. Maintaining a delicate balance between ROS and antioxidants is essential for cellular function. Excessive ROS can damage cellular macromolecules such as DNA, lipids, and proteins, ultimately leading to necrosis and apoptosis [32,33]. In both animal and cellular models, DHE staining confirmed that isorhamnetin reduced ROS production induced by isoprenaline. Our experiments showed that the serum MDA levels were significantly elevated compared to the control group, while the levels of SOD and GSH-PX were reduced in the model group. These results align with findings from other studies [34,35,36]. Both isorhamnetin and propranolol were able to reverse these changes. A large number of experiments have proved that oxidative stress is an important pathophysiological pathway for the occurrence and development of cardiovascular diseases [37,38]. Nrf2 plays a pivotal role in mitigating oxidative stress, with HO-1 serving as a key mediator in reducing myocardial oxidative stress injury. *Nrf2* activates the antioxidant response by translocating to the nucleus, where it binds to the promoter region of downstream genes, including *HO-1* [39]. In the present study, both animal and cellular experiments demonstrated that isorhamnetin increased the expression of Nrf2, HO-1, and NQO1 in damaged cardiomyocytes, thereby enhancing the cellular antioxidant response. These results suggest that isorhamnetin may protect against ISO-induced oxidative stress damage. Furthermore, oxidative stress is intricately linked to mitochondrial dysfunction [40,41,42]. Mitochondria are central to heart failure pathophysiology, influencing processes such as energy metabolism, oxidative stress, calcium homeostasis, and cell death [43,44,45]. The mitochondrial membrane potential (MMP) assay was conducted using JC-1. In normal cells with high mitochondrial membrane potential, a large amount of JC-1 in the mitochondrial matrix produces red fluorescence. In contrast, in apoptotic cells, the reduced MMP promotes the transfer of JC-1 to the cytoplasm, where it appears as green fluorescence [46]. Therefore, changes in MMP can be judged by changes in the fluorescence of JC-1. As shown in Figure 4G–J, the JC-1 mitochondrial membrane potential assay revealed that isorhamnetin effectively reversed ISO-induced mitochondrial damage, indicating its cardioprotective effects.

Transcriptomic RNA sequencing was performed on H9c2 cells, ISO-treated H9c2 cells, and isorhamnetin-pretreated H9c2 cells to elucidate the mechanism via which isorhamnetin alleviates isoprenaline-induced myocardial injury. GSEA was conducted on all enriched genes. Compared to the ISO group, isorhamnetin pretreatment significantly reduced the expression of several glycolytic metabolism genes, with *ENO1* identified as a key target for this downregulation. *ENO1*, a glycolytic enzyme involved in the ninth step of glycolysis, has been implicated in myocardial injury. Ji et al. demonstrated that serpina3c alleviates myocardial fibrosis post-MI by inhibiting *ENO1* transcriptional activation, thereby suppressing glycolysis [11]. Wu et al. further reported that elevated ENO1 expression leads to myocardial apoptosis, impaired collagen deposition, and systolic dysfunction [9]. Molecular docking revealed a strong affinity between isorhamnetin and ENO1, suggesting ENO1 as a direct target of isorhamnetin. PPARs—a key class of nuclear hormone receptors—regulate numerous cellular functions, with isoforms PPARα, PPARβ, and PPARγ playing pivotal roles in physiological processes [47]. PPARα, predominantly expressed in the myocardium, is a critical transcription factor in myocardial energy metabolism remodeling [48]. The activation of peroxisome proliferator-activated receptor γ coactivator 1α (*PGC-1α*) enhances the transcription of genes encoding enzymes involved in myocardial energy metabolism through paracrine signaling [49]. PGC-1α is a key molecular link between cellular fatty acid metabolism and mitochondrial biogenesis, which can prevent cardiac hypertrophy by regulating energy metabolism remodeling [50]. Immunoblotting experiments demonstrated increased ENO1 protein expression, alongside decreased PPARα and PGC-1α protein levels, in rat heart tissues exposed to ISO. These results suggest that the protective effect of isorhamnetin may be linked to ENO1 and glycolytic metabolism. It is hypothesized that PPARα may regulate isorhamnetin-mediated *ENO1* transcription, thereby modulating the myocardial response to isoprenaline-induced injury. Notably, during the onset of ISO-induced myocardial injury, key glycolytic enzymes were upregulated while ATP production decreased, indicating a metabolic shift from fatty acid metabolism to anaerobic glucose glycolysis [51]. This metabolic shift was reversed upon isorhamnetin pretreatment. To further confirm the role of ENO1, the enolase inhibitor AP-III-a4 (ENOblock) was applied, resulting in similar reductions in intracellular glucose and lactate levels and enhanced ATP production compared to isorhamnetin alone [44,52]. The combined use of both agents yielded more pronounced therapeutic effects, suggesting that isorhamnetin’s inhibition of ENO1 effectively suppresses glycolysis and improves energy metabolism.

In summary, ENO1 was identified as a key player in isoprenaline-induced myocardial injury in this study integrating transcriptomic analysis with in vitro and ex vivo cellular experiments. For the first time, it is demonstrated that isorhamnetin confers cardiovascular protective effects through the targeting of ENO1. Moreover, this research presents a novel perspective on isoprenaline-induced myocardial injury, highlighting the involvement of glucose metabolism. The inhibition of glycolysis and the correction of energy metabolism disturbances may underlie the cardiovascular protective effects of isorhamnetin. This study advances targeted therapy for cardiovascular diseases, broadens the understanding of isorhamnetin’s pharmacological targets, and provides a foundation for further exploration of its natural bioactive compounds. However, after the knockdown of ENO1, the effect on the cardioprotective effect of isorhamnoid was only verified in H9c2 cells, and no animal experiments were conducted. Further in vivo experimental verification is needed in the subsequent experiments.

## 5. Conclusions

In this study, we utilized molecular docking techniques and conducted in vivo and in vitro experiments to elucidate the protective effects of isorhamnetin against ISO-induced myocardial injury, as well as to investigate the potential mechanisms through which isorhamnetin exerts its effects. The results demonstrated that isorhamnetin ameliorated cardiac dysfunction and morphological damage, reduced the levels of serum markers of cardiac injury, and exerted cardioprotective effects by regulating oxidative stress and inhibiting apoptosis.

The results of the RNA sequencing study indicated that the primary mechanism through which isorhamnetin alleviated ISO-induced myocardial injury involved the glycolytic signaling pathway. Molecular docking and in vivo experiments suggest that isorhamnetin exerts its protective effects by inhibiting ENO1, activating the PPARα/PGC-1α signaling axis, reversing ISO-induced metabolic shifts in H9c2 cells, inhibiting glycolysis, and enhancing ATP release which, in turn, attenuates apoptosis and oxidative stress.

Despite the fact that this mechanism has not yet been exhaustively validated, the results obtained provide a solid research foundation for the utilization of isorhamnetin and a novel concept for the clinical prevention and treatment of ISO-induced myocardial injury.

## Data Availability

The data used in this study are confidential.

## Abbreviations

The following abbreviations are used in this manuscript:

ISO	Isoproterenol
ATP	Adenosine Triphosphate
ENO1	Recombinant Enolase 1
PPAR	Peroxisome Proliferator-Activated Receptor
PGC-1	Peroxisome Proliferator-Activated Receptor Gamma Coactivator 1
CVDs	Cardiovascular Diseases
EF	Ejection Fraction
FS	Fractional Shortening
CK-MB	Creatine Kinase-MB
DHE	Dihydroethidium
Nrf2	NF-E2-related factor 2
HO-1	Heme Oxygenase 1
NQO1	Recombinant NADH Dehydrogenase, Quinone 1
MDA	Malondialdehyde
SOD	Superoxide Dismutase
GSH-PX	Glutathione peroxidase
CCK-8	Cell Counting Kit-8
GSEA	Gene Set Enrichment Analysis
